# Humoral Immune Responses to *Burkholderia pseudomallei* Antigens in Captive and Wild Macaques in the Western Part of Java, Indonesia

**DOI:** 10.3390/vetsci7040153

**Published:** 2020-10-10

**Authors:** Vincentius Arca Testamenti, Rachmitasari Noviana, Diah Iskandriati, Michael H. Norris, Treenate Jiranantasak, Apichai Tuanyok, Aris Tri Wahyudi, Dondin Sajuthi, Joko Pamungkas

**Affiliations:** 1 Primatology Study Program, Graduate School of IPB University, Bogor, Jawa Barat 16128, Indonesia; atie@indo.net.id (D.I.); sajuthi@indo.net.id (D.S.); 2 Primate Research Center, IPB University, Bogor, Jawa Barat 16128, Indonesia; rachmitasari_iskandar@yahoo.co.id; 3 Spatial Epidemiology & Ecology Research Laboratory, Department of Geography, University of Florida, Gainesville, FL 32611, USA; mhnorris@ufl.edu; 4 Emerging Pathogens Institute, University of Florida, Gainesville, FL 32610, USA; treenate@ufl.edu (T.J.); tuanyok@ufl.edu (A.T.); 5 Department of Infectious Diseases and Immunology, University of Florida, Gainesville, FL 32610, USA; 6 Department of Biology, Faculty of Mathematics and Natural Sciences, IPB University, Bogor, Jawa Barat 16680, Indonesia; aristri2011@gmail.com; 7 Department of Clinics, Reproduction, and Pathology, Faculty of Veterinary Medicine, IPB University, Bogor, Jawa Barat 16680, Indonesia; 8 Department of Animal Infectious Diseases and Veterinary Public Health, Faculty of Veterinary Medicine, IPB University, Bogor, Jawa Barat 16680, Indonesia

**Keywords:** *Burkholderia pseudomallei*, ELISA, macaques, melioidosis, serosurveillance

## Abstract

*Burkholderia pseudomallei*, the Gram-negative bacterium which causes melioidosis, is a threat to human and a wide range of animal species. There is an increased concern of melioidosis in Indonesian primate facilities, especially following case reports of fatal melioidosis in captive macaques and orangutans. Our preliminary serosurveillance of immunoglobulin G (IgG) to *B. pseudomallei* lipopolysaccharide showed that a significant number of captive and wild macaques in the western part of Java, Indonesia, have been exposed to *B. pseudomallei.* To better characterize the humoral immune response in those animals, a panel of assays were conducted on the same blood plasma specimens that were taken from 182 cynomolgus macaques (*M. fascicularis*) and 88 pig-tailed macaques (*M. nemestrina*) reared in captive enclosures and wild habitats in the western part of Java, Indonesia. The enzyme-linked immunosorbent assays (ELISAs) in this study were conducted to detect IgG against *B. pseudomallei* proteins; alkyl hydroperoxide reductase subunit C (AhpC), hemolysin-coregulated protein (Hcp1), and putative outer membrane porin protein (OmpH). The performances of those immunoassays were compared to ELISA against *B. pseudomallei* LPS, which has been conducted previously. Seropositivity to at least one assay was 76.4% (139/182) and 13.6% (12/88) in cynomolgus macaques and pig-tailed macaques, respectively. Analysis of demographic factors showed that species and primate facility were significant factors. Cynomolgus macaques had higher probability of exposure to *B. pseudomallei*. Moreover, macaques in Jonggol facility also had higher probability, compared to macaques in other facilities. There were no statistical associations between seropositivity with other demographic factors such as sex, age group, and habitat type. There were strong positive correlations between the absorbance results of AhpC, HcpI, and OmpH assays, but not with LPS assay. Our analysis suggested that Hcp1 assay would complement LPS assay in melioidosis serosurveillance in macaques.

## 1. Introduction

Melioidosis, a disease caused by the saprophytic Gram-negative *Burkholderia pseudomallei*, is a threat to both human and animal health due to its considerably high mortality rate. The case fatality rate in humans is about 40% in highly endemic areas such as Thailand [1], 43% in Indonesia [2], and can reach 90% in patients with severe sepsis if untreated [1]. *B. pseudomallei* is classified as a Tier 1 Select Agent by the United States CDC. The bioterrorism potential of *B. pseudomallei* is related to its persistence in the environment, high prevalence of severe sepsis, and the ability to infect through aerosol, cutaneous, and ingestional exposures [3].

Melioidosis has been reported in Indonesia since 1929 [4,5]. Even though Indonesia has been known as an endemic area of melioidosis since the 1930s, cases remain underreported in the country [5]. Recently published case reports in human medicine include cases of four tsunami survivors in Banda Aceh [6] and three cases from a hospital in Makassar [5]. Based on a retrospective study by Tauran et al. [2], published melioidosis case reports in Indonesia do not represent the actual burden of the disease in the country. Many unpublished cases have been identified from hospitals in major cities such as Banjarmasin, Surabaya, Bandung, Jakarta, Yogyakarta, and Banda Aceh [2]. Published reports of melioidosis in the veterinary field in Indonesia are even more limited; those include cases in three cynomolgus monkeys exported to Britain [7], one pig-tailed monkey exported to the US [8,9], one cynomolgus monkey in IPB primate research center [10], and two orangutans in the facilities of Bornean Orangutan Survival Foundation [11,12].

As a response to the fatal melioidosis in cynomolgus monkey in IPB Primate Research Center [10], a surveillance was initiated to detect and characterize exposure to *B. pseudomallei* in macaques in several breeding facilities in the western part of Java, Indonesia. The concern arises because melioidosis cases in nonhuman primates are usually severe. Most of the reported spontaneous melioidosis cases in nonhuman primates (NHPs) resulted in either death during treatment or euthanasia [9,10,11,12,13,14,15,16]. The high mortality rate may greatly affect conservation efforts of protected NHP species and may also cause significant loss in primate centers working with macaques for biomedical purposes.

Melioidosis surveillance on a large population may encounter some obstacles. Melioidosis is called “the great mimicker” due to its non-characteristic and multisystemic lesions. Lesions may involve respiratory [17], genitourinary [18], skin and soft tissue [19], neurologic [20], or skeletal systems [21]. The challenge is even greater in veterinary medicine since prominent clinical manifestations of melioidosis may differ from one animal species to another. Culture is the gold standard in diagnosing melioidosis and is usually followed by confirmation assays such as the real-time PCR of type three secretion system (TTSS-1) [22], manual biochemical tests such as API^®^ 20NE), or automated system such as VITEK and matrix-assisted laser desorption/ionization time-of-flight (MALDI-TOF) [23,24]. Even though culture and bacterial identification are the best methods to diagnose the disease, those methods are neither cost-efficient nor effective in disease surveillance. Culture may also be challenging since the number of bacteria in blood samples of melioidosis patients may be as low as 1 CFU/mL [25]. Serological methods are generally preferred in disease surveillance in a large population since they are less time-consuming, less labor-intensive, and cost-effective. Serological assays for melioidosis have been developed, including latex agglutination test [26], indirect hemagglutination assay [27], and enzyme-linked immunosorbent assay (ELISA) [28,29,30].

The work presented here is an important step of melioidosis surveillance in macaque populations in Western part of Java, especially *M. fascicularis* and *M. nemestrina* in biomedical research facilities. The use of panel immunoassays allowed us to characterize the humoral immune response towards multiple recombinant proteins of *B. pseudomallei.* The serosurveillance was conducted by detecting immunoglobulins G towards *B. pseudomallei* alkyl hydroperoxide reductase subunit C (AhpC), hemolysin-coregulated protein 1 (Hcp1), and putative outer membrane porin protein (OmpH) by using ELISA. To evaluate the use of panel immunoassay in detecting exposure to *B. pseudomallei* in macaques, the results of recombinant protein assays were compared to the results of serosurveillance against *B. pseudomallei* LPS [31]. Demographic factors were also evaluated to support melioidosis mitigation plan in the studied primate facilities.

## 2. Materials and Methods

Assay development was conducted at the Emerging Pathogens Institute, University of Florida, Florida, USA. Animal sampling was conducted at primate facilities in the western part of Java, Indonesia; Jonggol, Darmaga, Lodaya, and Tinjil Island facilities. ELISAs were performed at the Primate Research Center, IPB University, Bogor, Indonesia.

### 2.1. Blood Sampling in Cynomolgus Macaques and Pig-Tailed Macaques

**Figure 1:**
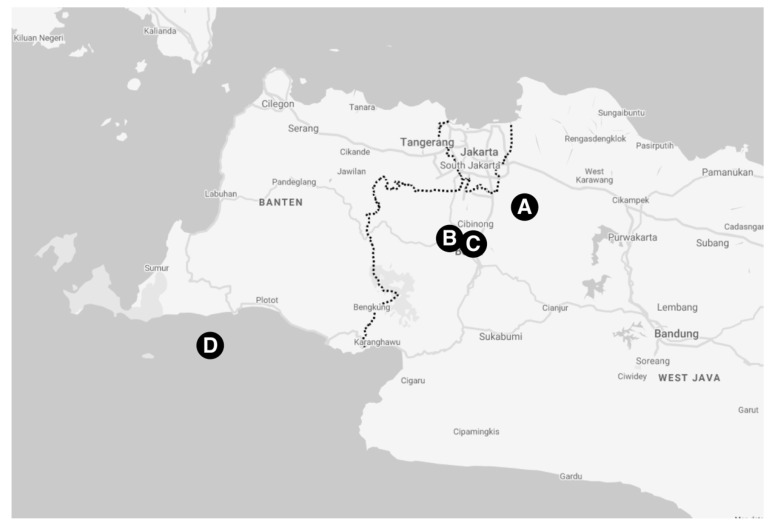
Map of western part of Java pointing NHP facilities in this study: Jonggol (A), Darmaga (B), Lodaya (C), and Tinjil Island (D).

**Figure 2:**
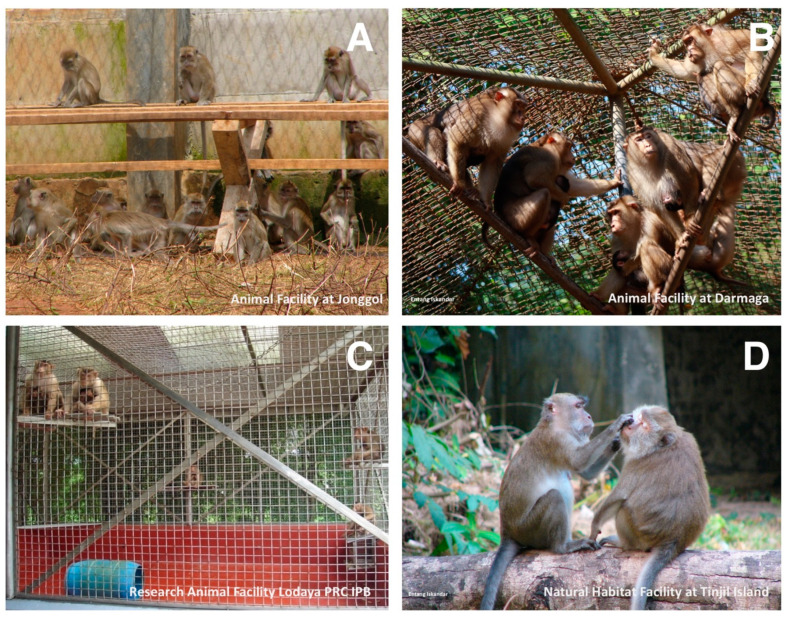
Cynomolgus macaques (*Macaca fascicularis,* **A** and **D**) and pig-tailed macaques (*Macaca nemestrina,* **B** and **C**). Jonggol, Darmaga, and Lodaya facilities (**A**–**C**) are captive enclosures, whereas Tinjil Island is a natural habitat facility (**D**). Photos courtesy of Entang Iskandar and RAFL PRC IPB.

All procedures in animals were carried out using protocol reviewed and approved by the Institutional Animal Care and Use Committee, Primate Research Center, IPB University, number ACUC IPB PRC-15-B0011 (approval date: 9 November 2015). From January to April 2016, a total of 270 blood samples were collected from 182 *Macaca fascicularis* and 88 *Macaca nemestrina* in selected breeding facilities in West Java (Figure 1): Jonggol (132/270), Darmaga (75/270), Lodaya (22/270), and Tinjil Island (41/270). Jonggol, Darmaga, and Lodaya facilities are captive enclosures that harbor both of *M. fascicularis* and *M. nemestrina*, whereas Tinjil island is a natural habitat breeding facility for *M. fascicularis* (Figure 2). Those primate facilities are managed by IPB Primate Research Center and PT. Wanara Satwa Loka. Captive macaques in this study were grouped by age, whereas wild macaques live in colonies with no separation. Prior to sample collection, all animals were anesthetized using ketamine (10 mg kg^−1^ body weight). Approximately 3 mL of blood was taken from the femoral vein of each animal. Following transport to Microbiology and Immunology Laboratory, Primate Research Center of IPB University, blood plasma was separated by centrifugation and was inactivated at 56 °C for 30 min.

### 2.2. Antigen Preparation

Recombinant proteins of AhpC (BPSL2096, ~21.37 kDa, accession number: WP_004192241), Hcp1 (BPSS1498, ~19.81 kDa, accession number WP_004525344), and OmpH (BPSL2150, ~18.13 kDa, accession number: WP_004199535) were produced as antigens for antibody detection. DNA sequences of *ahpC*, *hcp1*, and *ompH* were amplified from genomic DNA of biosafe *B. pseudomallei* strain Bp82 using primers that introduced *Sal*I and *Nde*I sites. Each PCR product was cut with these enzymes and separately ligated to pET21b cut with the same enzymes to generate C-terminal His-tagged proteins. Primers were designed to remove secretion signal sequences as predicted by the SignalP-5.0 server. Inserts were verified by digest and Sanger sequencing. Each vector plus an inserted DNA fragment was introduced into *E. coli* BL21(DE3).

The recombinant protein expression was performed by induction with 1 mM isopropyl β-d-1-thiogalactopyranoside (IPTG). Bacterial cell pellets were lysed, and histidine-tagged proteins were purified by using a HisPur Ni-NTA Spin Column (Thermo Massachusetts Scientific, Waltham, MA, USA) according to the manufacturer’s instruction. The eluates were desalted using Fisherbrand™ Regenerated Cellulose Dialysis Tubing (Fisher Scientific, Newington, NH, USA) according to the manufacturer’s instructions. Following the overnight desalting process at 4 °C, the soluble proteins were freeze-dried using FreeZone 4.5 L Benchtop Freeze Dry System (Labconco, MO, USA). The lyophilized proteins were resuspended in phosphate buffer saline solution (PBS).

The resuspended protein was quantified using PierceTM BCA Protein Assay Kit (Thermo Scientific, Chicago, IL, USA) according to the manufacturer’s instructions. SDS-PAGE and Coomassie blue staining were carried out to check the protein purification (Supplementary Materials, Figure S1). Western blot was performed by using mouse anti-6x-His-tag to visualize the His-tag harbored in the protein. The results of protein quantification and its purity by SDS-PAGE were evaluated to determine which eluate has the best antigen for immunoassay purposes.

### 2.3. Enzyme-Linked Immunosorbent Assay

ELISA assay was performed as follows. All wells in a single plate were coated with one antigen of interest, i.e., AhpC, Hcp1, or OmpH. ELISA plates were prepared by coating the 96-well microtiter plates with 100 μL of AhpC, Hcp1, or OmpH (1.25 μg/mL) at 4 °C overnight. Following the coating process, plates were washed again three times with PBS-Tween 0.05%. After blocking with 5% skim milk, the heat-inactivated plasma samples (1:1000 in blocking buffer) were added in duplicates to the ELISA plates and were incubated for 1 h at 37 °C. Then, plates were washed four times with PBS-Tween 0.5% and anti-monkey IgG (goat anti-monkey horseradish peroxidase conjugate, Sigma A2054, diluted at 1:1000 in blocking buffer) was added to all wells and incubated for 1 h at 37 °C. Following four washes, the chromogenic substrate 3,3′,5,5′-tetramethylbenzidine (TMB) was added to all wells to visualize the color development in the ELISA. Plates were incubated for 15–20 min in a dark environment. Thirty microliters of 1 N sulfuric acid was added to stop the reaction. Color development was measured using ELISA plate reader at 450 nm wavelength as measurement wavelength and 595 nm as the reference wavelength. The final absorbance value for each animal was calculated by taking the average measurement of the duplicates. The results of AhpC, Hcp1, and OmpH ELISA were compared to the results of ELISA toward *B. pseudomallei* lipopolysaccharide (LPS) as previously conducted [31]. ELISA plates coated with *B. pseudomallei* LPS were prepared and validated as previously described [30]. Due to unavailability of blood plasma sample from culture-confirmed melioidosis case, one plasma sample with high absorbance value was used consistently across all plates as control to account for inter-assay variability. Intra-assay variability was also calculated for each plate.

Since reports on exposure to *B. pseudomallei* in the macaques from the studied areas were limited, it was assumed that most of the animals have not been exposed to *B. pseudomallei*. Therefore, the seronegative population would follow a normal distribution curve. Cut-off values (λ_pos_) were set by three standard deviations (3 × σ) from the mean of the suspected negative population (μ) at 99.7% confidence interval. Statistical tests, including correlations between assays and associations between demographic factors such as species, sex, location, and age using logistic regression were performed in SPSS 16 (SPSS Inc., Chicago, IL, USA).

## 3. Results

### 3.1. Absorbance Results

**Figure 3:**
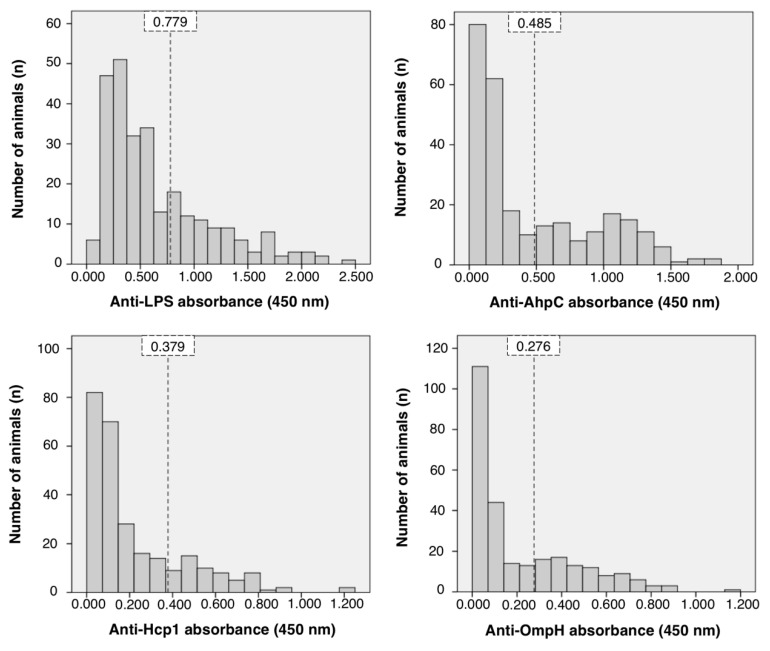
Distribution of absorbance values representing IgG against *B. pseudomallei* proteins; lipopolysaccharide (LPS), alkyl hydroperoxide reductase subunit C (AhpC), hemolysin-coregulated protein 1 (Hcp1), and putative outer membrane porin protein (OmpH). Vertical striped lines describe the cut-off value of each assay at a 99.7% confidence interval.

The absorbance values of ELISA against *B. pseudomallei* AhpC, Hcp1, and OmpH are presented in Figure 3, in comparison with the results of ELISA against *B. pseudomallei* LPS. For AhpC ELISA, mean of suspected negative population (μ) was 0.150, with standard deviation (σ) of 0.112, and a cut-of value (λ_pos_) of 0.485. For Hcp1 ELISA, the values were μ = 0.112 σ = 0.089, and λ_pos_ = 0.379. For OmpH ELISA, the values were μ = 0.074 σ = 0.067, and λ_pos_ = 0.276. For LPS ELISA, the absorbance values for the predicted seronegative population were distributed around μ = 0.338 and σ = 0.147, yielding a cut-off value (λ_pos_) of 0.779.

**Figure 4:**
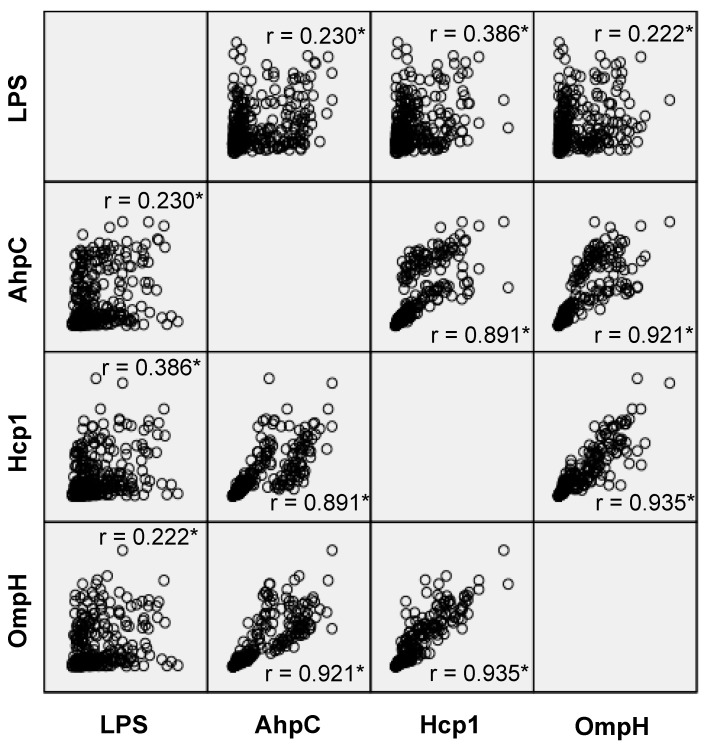
Scatter-plot relationship matrix between immunoglobulins G of anti-*Burkholderia pseudomallei* proteins; alkyl hydroperoxide reductase subunit C (AhpC), hemolysin-coregulated protein 1 (Hcp1), and putative outer membrane porin protein (OmpH), compared to the IgG of anti-*Burkholderia pseudomallei* lipopolysaccharide (LPS). The asterisks (*) indicate that correlation was significant at the 0.01 level.

The relationship between the four assays is described in Figure 4. There were significant correlations between each assay. Strong positive relationships were observed among AhpC, Hcp1, and OmpH assays, with Spearman’s correlation coefficients of 0.891, 0.921, and 0.935 for AhpC-Hcp1, AhpC-OmpH, and Hcp1-OmpH, respectively. However, such a strong correlation was not observed between LPS and the three other assays. Spearman’s correlation coefficients between LPS-AhpC, LPS-Hcp1, and LPS-OmpH were 0.230, 0.386, and 0.222, respectively.

### 3.2. Demography

Seroprevalence for each assay based on demographic factors is presented in Table 1. All cut-off values were set to give a 99.7% confidence interval. Statistical logistic regression showed that *M. fascicularis* and the Jonggol facility were significant demographic factors that had higher seropositivity to *B. pseudomallei*, as shown by AhpC, Hcp1, and OmpH assays (*p* < 0.005), but not by the LPS assay. No associations were observed between sex, age groups, or habitat types.

## 4. Discussion

Indonesia and many countries in the tropical region have a very suitable environment for *B. pseudomallei* [32]. In Indonesia, cases of melioidosis have been reported in both humans [2] and animals [7,8,9,10,11,12]. The number of reports is limited and interestingly, all veterinary cases of melioidosis in Indonesia (or in Indonesian animals exported abroad) were reported in nonhuman primates. All of them were animals reared under strict health monitoring, such as macaques in the IPB Primate Research Center, Bogor, Indonesia [10], cynomolgus macaques exported to the Britain [7], a pig-tailed macaque exported to the US [8,9], and orangutans in a Bornean Orangutan Survival Foundation rehabilitation and reintroduction programs in East Kalimantan [11] and Central Kalimantan, Indonesia [12]. The low number of melioidosis reports in veterinary medicine in Indonesia might be related to limited health monitoring and limited awareness of the disease.

The fatal septicemia case in a cynomolgus macaque in IPB Primate Research Center [10] raised a concern about whether other macaques in breeding facilities in western part of Java are exposed to the bacteria. To support our previous findings on high seropositivity towards *B. pseudomallei* LPS in the populations [31] and further characterize the humoral immune response in the macaques, antibody-detecting immunoassays towards multiple *B. pseudomallei* antigens were used in this study; alkyl hydroperoxide reductase subunit C (AhpC), hemolysin-coregulated protein (Hcp1), and putative outer membrane porin protein (OmpH).

This study showed that a significant number of the animals were seropositive to *B. pseudomallei* LPS, AhpC, Hcp1, and OmpH, but they did not show observable manifestations. Exposure to *B. pseudomallei* may occur through skin inoculation, ingestion, or inhalation. Soil and water have been proven to be important reservoirs of *B. pseudomallei* [33] and, therefore, it is likely that the macaques were exposed through skin inoculation or drinking unchlorinated water in those facilities. Even though *B. pseudomallei* cannot survive UV light [34], macaques may be exposed to the bacteria during the wet season which reveals deeper soil layers. Macaques in these breeding facilities have also been observed plucking grass and digging the soil, an activity that may increase the chance of being exposed to the bacteria, especially when they have open wounds due to aggression within the colony.

This serosurveillance showed that seropositivity in *M. fascicularis* is significantly higher compared to *M. nemestrina* (Table 1). In general, nonhuman primates are susceptible to melioidosis. Gorillas and orangutans are predicted to have a higher susceptibility among other NHP species, based on the high occurrence and severity of spontaneous melioidosis cases in those animals [2,15,35,36,37,38,39,40,41]. However, to the best of our knowledge, there have been no reports on susceptibility comparison between *M. fascicularis* and *M. nemestrina.* Host risk factors in animals are less well-defined than human risk factors. In humans, the predispositions of melioidosis infection include diabetes mellitus, thalassemia, aboriginality, male gender, soil/water exposure, renal disease, excessive alcohol consumption, and kava use [33].

The seroprevalence was also significantly higher in some facilities. The significantly higher seroprevalence in Jonggol and considerably high seroprevalence in Tinjil Island are most likely due to the environment in those breeding facilities. In Jonggol, animals are reared in a large group cage with soil flooring and an unchlorinated water source. In Tinjil Island, animals live in a secondary tropical rain forest. In those two facilities, contact between animals and soil is very high. In contrast, most of the animals in Darmaga and Lodaya are reared in group cages with either tile, rock, or concrete flooring. Our demographic factor analysis showed no statistically significant associations observed between seropositivity and other demographic factors such as sex, age group, and habitat type.

Surface antigens of *B. pseudomallei* such as LPS, O-polysaccharide component of the LPS, capsular polysaccharide (CPS), and Hcp1 have been developed as target antigens in detecting exposure to *B. pseudomallei* in human medicine [26,28,29,42]. In this study, we evaluated the use of multiple ELISAs (LPS, AhpC, Hcp1, and OmpH) in characterizing exposure to *B. pseudomallei* in macaques. LPS is a major outer membrane component of Gram-negative bacteria and is one of the main virulence factors of *B. pseudomallei* [43]. The LPS assay in this study utilized type A LPS, an antigen that was derived from *B. pseudomallei Bp82* Δ*wcb*, a capsular polysaccharide mutant of *B. pseudomallei* Bp82 [30]. Bp82 is an attenuated strain that has lost its virulence. The immunogenicity of the LPS used in this study was validated against sera of experimentally infected macaques [30].

A positive and strong correlation was seen among absorbance values in recombinant protein assays (AhpC, OmpH, and Hcp1, Figure 4). That strong correlation among recombinant protein assays suggests that the humoral immune response in monkeys responded to all those proteins at the same rate. However, such a strong positive correlation was not seen between absorbance values from LPS assay with recombinant protein assays. It is predicted that the LPS assay can detect recent exposures to the bacteria, whereas AhpC, Hcp1, and OmpH detect antibodies that are produced later. LPS is a strong T-cell independent antigen which can initiate antibody response without the help of T cells [44], but through its mitogenic activity that stimulates the production of polyclonal antibodies following Toll-like receptor (TLR) stimulation [45]. Immune response to LPS might be developed earlier and stronger when compared to antibodies toward AhpC, Hcp1, and OmpH. Therefore, the results of the LPS assay may not correlate strongly with other protein assays.

The strong positive correlation among the three recombinant protein assays may suggest that these recombinant protein assays may be performed interchangeably. However, it is predicted that the Hcp1 assay is the most specific among other assays in this study since Hcp1 is only expressed by *B. pseudomallei* and *B. mallei* in the *B. pseudomallei* complex. Protein sequences of AhpC and OmpH are present in other members of *B. pseudomallei* complex, such as *B. thailandensis, B. oklahomensis,* and *B. humptydooensis*, but the presence of those species in Indonesia remains unknown. Even though melioidosis cases should be confirmed by bacterial culture, serosurveillance is still beneficial in detecting and characterizing exposure to the bacteria, especially in large populations. Serosurveillance would give better results when performed by using a panel of assays. Based on the findings on this study, the use of Hcp1 assay which has a higher specificity and a moderately lower sensitivity may be beneficial to be paired with LPS assay, which has slightly lower specificity but higher sensitivity.

### Limitations

It remains unclear whether the seropositivity observed in this study is the result of an ongoing, asymptomatic infection or past resolved infections, as the animals did not show observable manifestations. Progress of the disease and seroconversion are beyond the scope of this study. We are also aware that for the LPS assay, the antibody may be produced following exposure to antigenically similar microorganisms, such as *B. thailandensis*. Even though the presence of *B. thailandensis* in Indonesia remains unknown, we included the use of more-specific assays based on recombinant antigens to increase the resolution of this serosurveillance. A study that analyzed the cross-reactivity across 1205 *B. pseudomallei* antigens by using microarray has shown that AhpC (BPSL2096) is considered as a *B. pseudomallei* antigen that has insignificant cross-reactivity and therefore, has a serodiagnostic value for melioidosis [46]. Other studies have also shown that Hcp1 assay has complementary serodiagnostic value when paired with OPS-assay, a derivative of LPS ([29,42,47]). The value of detecting antibody to *B. pseudomallei* OmpH protein has not been studied extensively, and here we discovered that the results from OmpH assay had a high correlation with AhpC and Hcp1 assays. This suggests that OmpH assay also has a serodiagnostic value for detection of antibody to *B. pseudomallei*.

Animal sampling in this study was conducted conveniently following the schedule of mandatory tuberculosis tests to comply with the 3Rs principle. Therefore, sample collection might not be representative of the whole population of animals in each breeding facility. Some age groups in this study may also be underrepresented due to their limited number in the population.

## 5. Conclusions

Nonhuman primates in this study demonstrated various levels of seropositivity towards *B. pseudomallei*, ranging from 13.3–87.9% in several breeding facilities. The number of seropositive animals in Jonggol is significantly higher, and it is predicted that the main contributing factor is the high exposure to the soil in that facility. Based on the high correlation between recombinant protein assays (AhpC, Hcp1, and OmpH), we suggest that the use of LPS assay to be paralleled with one of those recombinant protein assays (preferably Hcp1) for future serosurveillance of melioidosis. This serological study in macaques needs to be followed by culture of both environmental samples from primate enclosures and clinical samples from seropositive animals if the animals develop symptoms of melioidosis.

## Figures and Tables

**Table 1:** Seroprevalence in macaques by demographic factors.

Demographic Factors	n	N of Seropositive Animals in Each Assay (%) ^1^				Overall Seropositivity ^3^ (%)
		LPS ^2^	AhpC	Hcp1	OmpH	
Animal species						
M. fascicularis *	182	72 (39.6)	101 (55.5)	58 (31.9)	87 (47.8)	139 (76.4)
*M. nemestrina*	88	11 (12.5)	0 (0.0)	0 (0.0)	1 (1.1)	12 (13.6)
Sex						
Female	171	59 (34.5)	54 (31.6)	47 (27.5)	53 (31.0)	91 (53.2)
Male	99	24 (24.2)	47 (47.5)	11 (11.1)	35 (35.3)	60 (60.6)
Location						
Jonggol *	132	49 (37.1)	100 (75.7) *	58 (43.9) *	87 (65.9) *	116 (87.9)
Darmaga	75	9 (12.0)	0 (0.0)	0 (0.0)	1 (1.3)	10 (13.3)
Lodaya	22	3 (13.6)	0 (0.0)	0 (0.0)	0 (0.0)	3 (13.6)
Tinjil	41	22 (53.7)	1 (2.4)	0 (0.0)	0 (0.0)	22 (53.7)
Age						
1–2	10	1 (10.0)	0 (0.0)	0 (0.0)	0 (0.0)	1 (10.0)
2–3	2	0 (0.0)	0 (0.0)	0 (0.0)	0 (0.0)	0 (0.0)
3–4	52	18 (34.6)	37 (71.1)	18 (34.6)	29 (55.8)	42 (80.8)
4–5	88	23 (26.1)	25 (28.4)	19 (21.6)	31 (35.2)	45 (51.1)
5–6	93	34 (36.5)	29 (31.2)	21 (22.6)	27 (29.0)	55 (59.1)
6–7	11	0 (0.0)	0 (0.0)	0 (0.0)	0 (0.0)	0 (0.0)
7–8	8	4 (50.0)	0 (0.0)	0 (0.0)	0 (0.0)	4 (50.0)
9–10	4	3 (75.0)	0 (0.0)	0 (0.0)	0 (0.0)	3 (75.0)
10–11	2	0 (0.0)	0 (0.0)	0 (0.0)	1 (50.0)	1 (50.0)
Habitat ^4^						
Captivity	229	61 (26.6)	100 (43.7)	58 (25.3)	88 (38.4)	129 (56.3)
Wild	41	22 (53.6)	1 (2.4)	0 (0.0)	0 (0.0)	22 (53.7)

^1^ LPS: lipopolysaccharide, AhpC: alkyl hydroperoxide reductase subunit C, Hcp1: hemolysin-coregulated protein 1, OmpH: putative outer membrane porin protein. ^2^ Based on absorbance values in the previous investigation [31]. ^3^ Positivity to at least one assay. ^4^ Captivity (Jonggol, Darmaga, and Lodaya) and wild habitat (Tinjil Island). * Significantly associated with seropositivity (*p* < 0.005).

## Supplementary Materials

The following are available online at https://www.mdpi.com/2306-7381/7/4/153/s1, Figure S1: Coomassie blue staining of AhpC (a), Hcp1 (b), and OmpH (c) following Ni-NTA purification.
