# Supplementary material for: Humoral Immune Responses to Burkholderia pseudomallei Antigens in Captive and Wild Macaques in the Western Part of Java, Indonesia
Source: Vet Sci. 2020 Oct 10;7(4):153. doi: 10.3390/vetsci7040153 (PMC7712568; doi:10.3390/vetsci7040153)
Supplement: Supplementary file 1 [file vetsci-07-00153-s001.pdf]

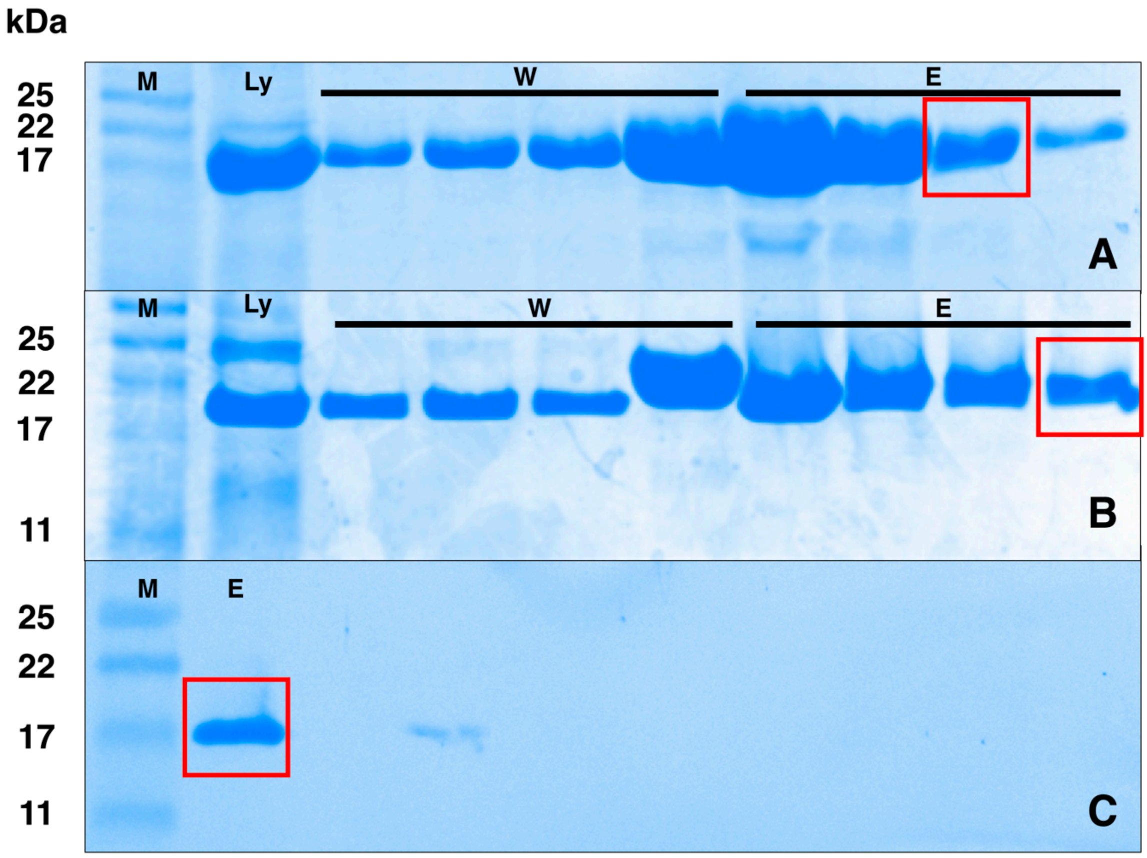

Figure 1. Coomassie blue staining of AhpC (a), Hcp1 (b), and OmpH (c) following Ni-NTA purification. The eluates used for ELISA plate coating are marked with red rectangles. M: protein ladder, Ly: cell lysate, W: wash flow through, E: eluate
